# European Public Health News

**DOI:** 10.1093/eurpub/ckad106

**Published:** 2023-08-01

**Authors:** Marieke Verschuuren, Maaike Droogers, Damir Ivanković, Hans Henri P Kluge, Robb Butler, Katrine Habersaat, Anthony Staines, Floris Barnhoorn

**Affiliations:** EUPHA; EUPHA; EUPHA; WHO Regional Office for Europe; WHO Regional Office for Europe; WHO Regional Office for Europe; EPH Conference; EPH Conference

In this edition of the European Public Health News, the EUPHA office celebrates the European Public Health Week as a successful event and fixed value for public health in Europe, while we also mourn the loss of Richard Horst Noack, EUPHA past president. An obituary on this remarkable public health colleague, written by Dorner and Zeegers Paget, is published as an editorial in this edition of the European Journal of Public Health. The WHO Regional Office for Europe puts the spotlight on behavioural and cultural insights, and how WHO-Euro and its member states will work together to harness these in a systematic and evidence-based manner to tackle our most pressing health challenges. Finally, you will find in this section an update on the plenary programme of the European Public Health Conference in November this year in Dublin.

## Figures and Tables

**Figure 1:**
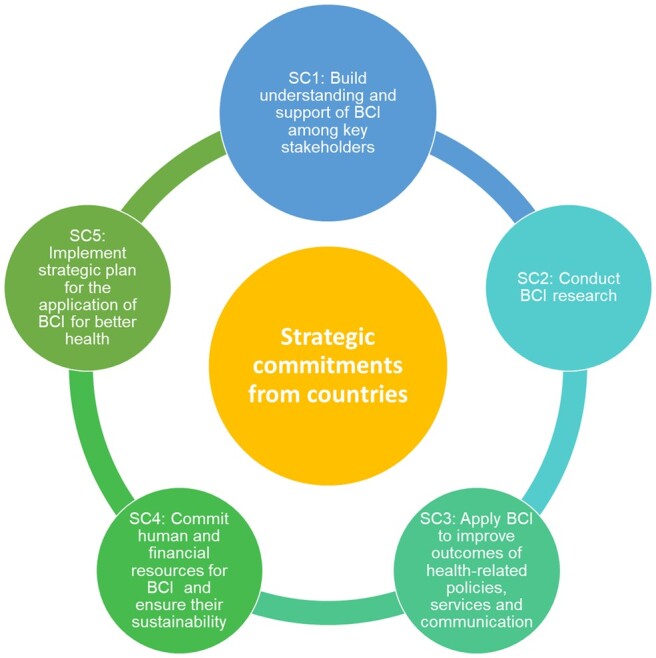
Strategic commitments (SC) for behavioural and cultural insights (BCI) adopted by countries in Europe and Central Asia on 13 September 2022

## The European Public Health Week: a success story of joining forces for a common cause

VerschuurenMarieke
EUPHA
DroogersMaaike
EUPHA
IvankovićDamir
EUPHA

From 22 to 26 May 2023, the fifth edition of the European Public Health Week (EUPHW) took place. The EUPHW is an initiative of EUPHA. Its aim is to raise awareness about public health, stimulate positive action and promote collaboration among the public health community in Europe. How it works is that EUPHA provides a central communication platform and communication tools, while all organizations and individuals that want to contribute to the aims of the EUPHW and subscribe to EUPHA’s values can organize events under the EUPHW umbrella. The EUPHW is supported by WHO Regional Office for Europe. The coordination work for most of the EUPHW editions so far, including the 2023 edition, was co-funded by the European Union through an operating grant.

The Week always has an overarching theme as well as five daily themes, representing important and topical public health issues. The overarching theme for the EUPHW 2023 was ‘Public health: global issues, local actions’, while the daily themes centred around mental health, diversity in health, access to care, chronic diseases and global health emergencies preparedness and response.

Since its inception in 2019, the EUPHW has been very successful. While in the first 2 years around 145 events were organized, in 2021 and 2022 there were over 200. This trend was continued in 2023, when a total of 224 events were organized during the week in 21 languages across 39 countries in Europe and beyond. Events are a mixture of in-person, online and hybrid events and can range from local events like walking in the park or health promotion activities at schools to online serious games and webinars with a global reach.

It is heart-warming to see the public health community rise the occasion year after year and make an effort to keep public health on the agenda. Through their contributions, the EUPHW successfully brings together numerous stakeholders, including public health professionals, policymakers, researchers and the general public. The Week also facilitates networking opportunities through various means, including virtual forums, discussion panels and interactive sessions. Participants have the chance to connect with experts from different countries and share their experiences, ideas and research findings. And with more than half of all the events in 2023 being organized jointly by multiple organizations and/or institutions, the collaborative nature of the EUPHW is clearly manifested.

In 5 years that the EUPHW has been organized, the initiative by now is well-established as a fixed value for public health in Europe. The growing interest for the Week shows that EUPHA’s members and partners feel a continued need to actively make the case for healthy populations in Europe and beyond. The EUPHA office thanks all who have contributed to the success of the EUPHW so far and we look forward to many more interesting, enthusing and impactful editions!

## Behaviours lie at the heart of critical health challenges—and hold the key to solving them

KlugeHans Henri P
WHO Regional Office for Europe
ButlerRobb
WHO Regional Office for Europe
HabersaatKatrine
WHO Regional Office for Europe

Health systems today face unprecedented pressures: rising costs related to ageing populations and a soaring demand for services, as well as medicine shortages, and an overstretched health and care workforce.

In the face of these challenges, no opportunity for more effective and people-centred health and care services should be left untapped, and we must apply what evidence has proved works with renewed rigour. Using behavioural and cultural insights for health policies, services and communications offers such an opportunity.

We know that behaviours—from the use and prescribing of antibiotics, uptake of vaccination, alcohol and tobacco use, physical inactivity, to adherence to treatment and rehabilitation plans—lie at the heart of our most pressing public health challenges. Indeed, of all the factors affecting our health and well-being, behaviours have the greatest influence^1^^,^^2^.

Robust methods, theoretical models and rich evidence exist in this field that can help inform evidence-based approaches to health behaviours. Knowing this, however, has so far not translated into sustainable investment by health organizations and authorities, and this field remains largely underutilized, underexplored and underfinanced in public health.

Newly reported data from public health authorities in the WHO European Region show that although many countries have been exploring the determinants of certain health behaviours and have used these insights to inform their work, this is rarely done systematically. In addition, the majority of countries report not having sustainable funding, human resources, effective stakeholder relations or national strategic plans in place to support this work.

Fortunately, light is on the horizon: on 13 September 2022, 53 WHO Member States in Europe and Central Asia unanimously adopted a resolution and action framework on the use of evidence-based approaches to health behaviours^3^^,^^4^ and this has inspired a global resolution in May 2023. With these resolutions, the WHO Regional Office for Europe is accelerating the use of behavioural and cultural insights (BCI) for health across Europe and Central Asia, under the European Programme of Work, and its flagship initiative on the same topic.

Behavioural and cultural insights involve gaining a nuanced understanding of the factors that affect health behaviours and tailoring health services, policies and communications to the needs and circumstances of the people and communities affected. Evaluation plays a critical role in this, to document the impact of these interventions using robust methodology such as randomized controlled trials, as well as exploring their broader influences and acceptability before further roll-out. Together, using insights and evaluation ensure that the most effective interventions are applied and provide a cost-effective return on investment.

There are many compelling examples of the impact that behavioural and cultural insights can make on health. These include documented changes in healthy food purchases through nutritional front-of-pack labelling; increasing vaccination through addressing community-specific barriers; decreasing the consumption of high-sugar drinks by 30% using a new tax design; improving equal access to care through intercultural mediation; boosting response rates to hospital appointments by the 20% of non-responders through behaviourally informed invitation letters and reducing antibiotic prescriptions and corresponding spending in the UK by GBP 93 000 through sending targeted letters to general practitioners^5^^,^^6^.

With the regional resolution, national health authorities have made five ambitious commitments covering the next 5 years (figure 1). They have agreed to invest more human and financial resources, develop national plans, work with stakeholders as well as conduct insights research, and use this to inform health policies, services and communications. This work will be undertaken following principles of equity, participation, and above all, people-centeredness.

While the regional and global resolutions are significant advances, they cannot stand alone. To support their implementation, WHO/Europe is working with Member States in four areas. First, to build capacity among national health authorities in using behavioural and cultural insights evidence and methodology, through face-to-face and online training opportunities. Second, to develop guidance and policy recommendations, as well as compile and disseminate new evidence and best practices on the effective application of behavioural and cultural insights. Third, to strengthen engagement, collaboration and coordination between national, regional and international organizations and non-state actors and facilitate peer-to-peer and community-of-practice activities and dialogue. Fourth, to advocate for greater investment in behavioural and cultural insights for health among national decision-makers and international donors and partners.

One year on from the adoption of the regional resolution, WHO/Europe will convene a meeting on behavioural and cultural insights for health in Copenhagen, Denmark to advance its implementation and strategic commitments. BCI stakeholders from public health authorities across the WHO European Region will come together to exchange case examples, discuss barriers and drivers to a range of health challenges and explore progress in integrating BCI for health into health systems.

Given the crisis of trust in our health system infrastructure and the numerous threats to health we face, we must use every tool at our disposal to improve health, well-being and equity. Behavioural and cultural insights represent an untapped and powerful opportunity to do so. We look forward to advancing the commitments made in the regional resolution and action framework, harnessing behavioural and cultural insights in a systematic and evidence-based manner to tackle our most pressing health challenges. One billion people in the WHO European Region stand to benefit.

## Our Food, Our Health, Our Earth: A Sustainable Future for Humanity

StainesAnthony
EPH Conference
BarnhoornFloris
EPH Conference

16th European Public Health Conference

8–11 November 2023

Convention Centre Dublin (The CCD), Dublin, Ireland

Over the next decade, choices will be made which will determine the future of our advanced technological civilization. We now have no way out in terms of climate emergency. The global climate is rapidly deteriorating. We know what to do, to deliver a future for our society, but it’s less clear that we will do it. Another COP will be organized in December this year to find ways to pull humanity back from the climate cliff. Building a future, any kind of future, for us and our children, demands a new attention to sustainability. Prior to COP, at the EPH Conference in Dublin, we will open part of this discussion, with a focus on sustainability, food and health. We will look specifically at global health, the use of digital tools, equity in health and the necessary staffing and skills to provide good care. We will, in line with the overall conference theme, look in depth at the climate emergency, and very specifically at the human food supply.

### Plenary programme

The EPH Conference plenary programme kicks off with a session called Transitioning to more sustainable food systems that support health and wellbeing. The session is organized by EuroHealthNet, the not-for-profit partnership of organizations, institutes and authorities working on public health.

What measures are urgently needed to ensure more sustainable food systems and to improve health and well-being across Europe? What is the role of health professionals in helping to achieve this critical shift. Food is central to our health and well-being, yet many aspects of our food systems have become unhealthy and unsustainable. Our food systems today contribute significantly to the environmental crises we are facing. The consumption of low-quality diets is a major contributor to the burden of NCDs in Europe. Food systems have also contributed to widening health inequalities across Europe, as parts of the population have little access to healthy and sustainable food. Major external shocks such as COVID-19 and the Russian war against Ukraine have reflected the vulnerability of our current systems of food production and distribution, as experienced through growing levels of food inflation, food insecurity in Europe and even more so in other parts of the world.

Ambitious intersectoral approaches are needed to address current contradictions in our food systems. Public health professionals need to step up their action to highlight the impacts of unhealthy food consumption, advocating and engaging in political debates. Promising policies at the EU level, such as the Farm to Fork Strategy, Sustainable Food Law and on Food Security, set out planned measures to make food systems more environmentally friendly, fair and healthy. Ensuring that such policies are actually implemented and that environmental, health and social considerations are valued in equal measure as economic ones, remains however a major challenge.

Speakers include Tim Lang, City, University of London, United Kingdom; Gunhild A. Stordalen, EAT Foundation; Holly Cairns, Ireland, TD for Cork South-West; Anant Jani, Oxford University, United Kingdom.

Other plenary sessions include:

- Non-communicable diseases: risk factors and commercial determinants, organized by EUPHA and European Observatory on Health Systems and Policies.
- Building the future One Health workforce, organized by ASPHER and EHMA.
- On the path to an equitable and sustainable digital future for European health systems, organized by WHO Regional Office for Europe.
- Health security, organized by DG SANTE, European Commission.

Names of speakers and moderators will be announced shortly on our website (https://ephconference.eu/2023-plenary-programme-535). Read more about the plenary programme here.

We look forward to seeing you all in person, in Dublin.
